# Beyond Usual Care: A Multidisciplinary Approach Towards the Treatment of Obstructive Sleep Apnoea

**DOI:** 10.3389/fcvm.2021.747495

**Published:** 2022-01-05

**Authors:** Miuni Athauda Arachchige, Joerg Steier

**Affiliations:** ^1^CHAPS, Faculty of Life Sciences and Medicine, King's College London, London, United Kingdom; ^2^Lane Fox Unit/Sleep Disorders Centre, Guy's and St Thomas' NHS Foundation Trust, London, United Kingdom

**Keywords:** CPAP (continuous positive air pressure), hypoglossal nerve stimulation (HNS), transcutaneous electrical stimulation (TES), mandibular advancement device (MAD), non-CPAP treatment of sleep apnea

## Abstract

Obstructive Sleep Apnoea (OSA) is common and characterised by repeated apnoeas and hypopnoeas while asleep due to collapse of the upper airway. OSA can have a significant impact on physical and mental health and, when left untreated, is associated with increased risk of developing cardiovascular ill health. Besides cardiorespiratory implications excessive daytime sleepiness, morning headaches, limited memory function and lack of concentration are some further symptoms caused by OSA. Continuous Positive Airway Pressure (CPAP) therapy is the evidence-based treatment to maintain upper airway patency in patients with moderate to severe OSA. Proper adherence to CPAP therapy successfully abolishes nocturnal apnoeas and hypopnoeas, and diminishes consequences of uncontrolled OSA, such as treatment resistant hypertension. However, long term adherence to CPAP remains an unresolved limitation of this method. Although alternatives to CPAP therapy may be less efficacious, there is a variety of non-CPAP treatments that includes conventional lifestyle advice, postural advice, the use of mandibular advancement devices (MADs), surgical treatment options, such as uvulopalatopharyngoplasty, tonsillectomy, or maxillomandibular advancement, and the use of electrical stimulation of the upper airway dilator muscles. Hypoglossal Nerve Stimulation is available as an invasive (HNS) and a transcutaneous (TESLA) approach. For the management of “difficult-to-treat” patients with OSA, particularly in those in whom first line therapy proved to be unsuccessful, a multidisciplinary team approach may be helpful to incorporate the available options of non-CPAP therapy and provide appropriate choices. Symptom control, patient-related outcome measures and long-term cardiovascular health should be prioritised when choosing long-term therapies to treat OSA. The inclusion of patients in the choice of successful management options of their condition will facilitate better long-term adherence. Advancing clinical trials in the field will further help to resolve the relative lack of evidence for effective non-CPAP methods.

## Introduction

Sleep Apnoea is a common sleep-related breathing disorder, causing pauses in breathing during sleep. There are different types of Sleep Apnoea, including Obstructive Sleep Apnoea (OSA), Central Sleep Apnoea (CSA) and Mixed Sleep Apnoea. However, OSA is the most prevalent type, affecting an estimated 1.5 million adults in the UK alone, with up to 85% of the affected patients being undiagnosed (1).

OSA is caused by an obstruction of the upper airway during sleep, leading to recurrent collapse of the pharyngeal space (2). This results in either partial blockage (hypopnoea) or complete obstruction (apnoea). The resulting combination of intermittent hypoxia, increased airway resistance and CO_2_ changes result in sleep fragmentation. This leads to reduced slow-wave sleep and less time in REM sleep. Sleep fragmentation can further lead to both reduced sleep quantity and quality of sleep.

Patients with OSA may have low levels of consciousness at night (3), and diagnosis is often made upon observation by others, or patient's complaints of daytime fatigue and tiredness. OSA is associated with many problems, including affecting memory, daytime vigilance, causing morning headaches and excessive daytime sleepiness, and it is linked with increased cardiovascular risks (4).

Factors associated with OSA include certain a) anatomical features (1), including a large neck circumference, a set-back lower jaw, enlarged tonsils, macroglossia, and abnormal shapes of the upper airway, b) obesity (1–3), c) gender, with men being more affected than women, and d) age (1, 2, 5). A low arousal threshold, reduced neuromuscular tone and complete the approach toward characterisation of physiological traits in OSA (6).

## Impact on Health

OSA can cause excessive daytime sleepiness (EDS), independent of severity of the syndrome, impacting an individual's concentration during work and activities of daily life. It also has a substantial effect on memory as unfragmented sleep is essential for the development and consolidation of semantic memory (7). In addition, depression is a common symptom in untreated OSA (8).

Furthermore, those with untreated OSA are at an increased risk of having a motor vehicle accident (8), so those with job roles involving driving, e.g., professional drivers, are at particular risk if they suffer from undiagnosed OSA. The current estimate for the prevalence of OSA in HGV drivers is over 15% (1). Similar caution should be taken toward untreated OSA when handling heavy machinery or undertaking manual labour tasks that could put the patient of others at risk.

Untreated OSA increases the risk of cardiac conditions, particularly in moderate-severe disease, such as hypertension and heart disease. The recurrent apnoeas and hypopnoeas result in intermittent hypoxia, thus increased the sympathetic tone and the cardiac output. As a consequence, both the heart rate and the blood pressure increase, which can contribute to hypertension and increased cardiac stress (9). Cardiovascular disease accounts for 42% of deaths in people with OSA, compared to 26% in people without the condition (1).

It is estimated that up to 70% of those with type 2 diabetes have a form of sleep-disordered breathing (1), although it is possible that this is due to the close features of similar phenotypes leading to OSA and diabetes via the joint factor of obesity.

## Diagnosis

There are classic symptoms that patients suffering from OSA present with, such as snoring, fragmented sleep due to choking or gasping episodes, witnessed apnoeas or hypopnoeas by the partner, family or friends, and EDS, as measured by the Epworth Sleepiness Scale (10). Therefore, if the history is indicative of OSA and the patient has notable risk factors, the clinician should carry out further tests to confirm the diagnosis (11).

These include a) a physical exam to look for contributing factors or conditions, including an oral inspection, and the measurement of height, weight and neck circumference, b) blood tests, including a full blood count, thyroidal function tests, iron levels/ferritin and HbA1c, and c) considering to undertake a sleep study. For the latter, home- vs. inpatient sleep studies and level of quality (pulse oximetry, limited channel respiratory polygraphy, or polysomnography) should be discussed with the patient and chosen according to appropriateness (12).

The results of an overnight monitoring are then analysed to calculate the Apnoea Hypopnea Index (AHI), or in the case of a pulse oximetry the Oxygen Desaturation Index (ODI). These indices measure the number of episodes (of paused breathing) an individual experiences while asleep. These indices are also used to classify the severity of the OSA:

- Mild OSA: AHI 5–15 events x hour^−1^
- Moderate OSA: AHI 15–30 events x hour^−1^
- Severe OSA: AHI 30 or more events x hour^−1^ (1, 3, 13).

## Treatment

The advent of CPAP therapy in the 1980's provided a highly efficient treatment for patients with OSA (14) and triggered the opening of many clinical sleep services. However, long-term adherence to CPAP therapy is limited, with studies using “big data” reporting about 75% at 3-months (15, 16) It is therefore time to re-think a widely practised “one-size-fits-all” approach. The recently published NICE guidelines on OSA (17) have shifted the focus on the use of Mandibular Advancement Devices (MAD), while the recent European Respiratory Society (ERS) guidelines on non-CPAP therapy (18) mention further therapeutic choices and promote future research to develop the existing evidence base. The wider involvement of different specialties would favour “patient's choice.”

### Lifestyle/Primary Care, Dietitian, Metabolic and Bariatric Services, and Sleep Experts

Lifestyle with regular sleep hygiene, routine and dietetic advice needs to be at the forefront of any consultation when the diagnosis has been confirmed. OSA is more prevalent in obese individuals, and weight loss has the potential to reduce the severity of OSA, in some cases leading to a cure (19). In some cases, a possible referral to the bariatric team may be indicated. Increased physical activity, reduction of alcohol and caffeine containing drinks should also be discussed.

### Postural Advice/Primary Care and Sleep Expert

Positional therapy promoting sleeping in a lateral posture instead of a supine position, or sleeping with the upper body being elevated is another way to improve OSA severity, and in postural OSA abolish the problem (3). In non-supine posture, the gravitational impact on the structures of the upper airway is less likely to be disadvantageous (20). However, positional therapy is more effective in younger and less obese patients, and not appropriate for all OSA sufferers (21).

### Continuous Positive Airway Pressure/Respiratory Expert

CPAP therapy is the current recommended best available treatment for patients with moderate or severe OSA. CPAP requires to sleep with an interface, either a nasal or oronasal mask that the patient places over their airway while asleep; the mask is connected with a tube to a bedside machine that is typically powered by the mains and that produces compressed room air which then enters the patient's upper airway and prevents it from collapsing. It requires, however, to use the treatment everytime when the patients go to sleep. Proper adherence to CPAP therapy abolishes nocturnal apnoeas and hypopnoeas, intermittent hypoxia and sleep fragmentation, and leads to significant improvements in the patient's quality of life, including reduction in EDS and other consequences that may have been contributing to by uncontrolled OSA (e.g., hypertension) (22), in the majority of those treated (1). During the Covid-19 pandemic, CPAP therapy has been largely provided in a remote manner, as it is deemed to be an aerosol-generating procedure (APG). Pressurisation has frequently delayed until patients had access in their own home with remote guidance and monitoring. Adherence data are currently being reviewed, as it is important to understand the impact of remote pathways compared to the conventional way of CPAP setups.

Despite CPAP being successful in controlling airway patency when asleep, long-term adherence remains problematic. Adherence rates differ significantly, depending on the definition of adherence used and the cohorts of patients followed, but generally seem to range from 30 to 60% (23). Low adherence may be caused by many factors, including poorly fitted masks, problems with nasal and oral airway dehydration, nasal bleeding or throat irritation, but also individual health beliefs, self-image and partners' perception (24). However, educating the patient on the benefits of CPAP, such as through nurse- or physiologist-led courses, can help to significantly improve adherence (1).

Another factor is that CPAP does not cure OSA itself, but rather acts as a treatment to suppress it, allowing the patient to continue to sleep undisturbed. However, some patients may look for and prefer a more permanent solution to their problem by seeking other medical treatments or, potentially, surgery (21).

The National Institute of Health and Care Excellence (NICE) guidelines recommend CPAP therapy as the first-line treatment for moderate or severe obstructive sleep apnoea syndrome. Currently, its use it recommended in mild sleep apnoea only if the symptoms experienced cause a significant impact on their sufferer's quality of life or if other interventions/treatments have failed (24). However, the latest published NICE guideline proposes CPAP as treatment for mild OSA as well (17). It has, however, been established that certain phenotypes may benefit more from CPAP than others, such as those younger than 60 years, those with uncontrolled blood pressure at baseline or those with severe oxygen desaturations (24, 25).

Behavioural interventions to improve CPAP usage have shown interesting results with possible improvements in adherence, although there remain controversial data as to whether long-term compliance can be improved. However, cognitive behavioural therapy (CBT) may help with desensitisation and, particularly in patients with comorbid insomnia (COMISA), may be a suitable means to improve long-term compliance. What seems to be important is that early adherence practise predicts long-term adherence, and in this context it is important to acknowledge that time invested with the patient at initiation of the treatment is essential.

### Non-CPAP Therapies Provided With the Sleep Centre

#### Mandibular Advancement Device/Dentist

The MAD is an oral appliance, either over-the-counter, semi-bespoke or customised by a dentist mouthpiece that shifts the lower jaw forwards, opening the pharyngeal airway and improving upper airway patency while asleep (3). They are currently recommended by NICE for those who snore, patients who experience mild OSA (without any effect on daytime alertness), or those unable to tolerate CPAP (24). Overall, MADs may be slightly less effective than CPAP with regards to improvement of the AHI, but they have a similar efficacy with regards to symptoms, and, as they may be better tolerated in the long-term, they offer a good treatment alternative, particularly in the mild OSA cohort (21).

#### Hypoglossal Nerve Stimulation/ENT Surgeons

Hypoglossal nerve stimulation is an emerging treatment for OSA. There are different methods of applying electrical stimulation to the hypoglossal nerve. The invasive approach involves the implantation of a neurostimulator in an infraclavicular subcutaneous pocket which delivers an electric current to the distal branch of the hypoglossal nerve, thus causing the genioglossus muscle in the tongue to contract, preventing the tongue from prolapsing backwards and causing airway obstruction during sleep (26). Overall, trial results revealed that the median AHI score at 12 months decreased by 68% (26). However, the invasiveness of the approach, costs and potential side effects need to be considered (26, 27). Although NICE have tested the safety and efficacy of this procedure in the UK, it is only recommended after explicit patient selection and providing the procedure is conducted by clinicians specialised in OSA management (28).

#### Transcutaneous Electrical Stimulation in Sleep Apnoea/Sleep Expert, Research Trial

**Figure 1:**
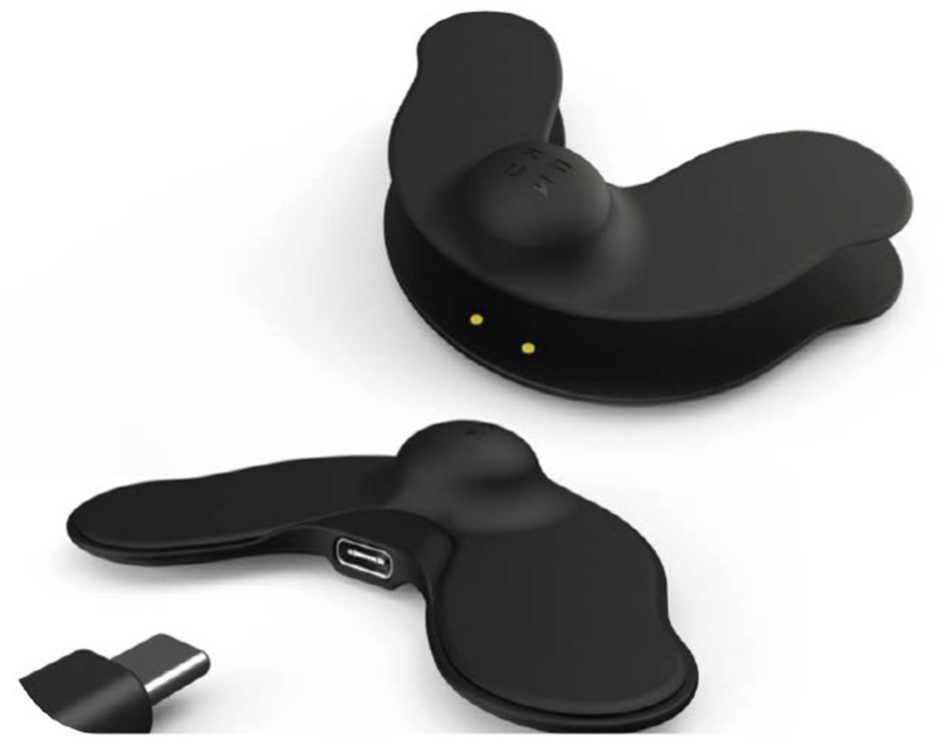
The TESLA home device with its components, stimulator, USB connector, patch. With friendly permission of Morgan IAT (ZEUS™, Petersfield, UK).

A non-invasive approach to deliver electrical current to the hypoglossal nerve is TESLA (29). It consists of transcutaneous patches that can be applied to the skin in the submental area, allowing stimulation of the upper airway dilator muscles via the hypoglossal nerve, this includes the genioglossus (Figure 1). Upper airway dilators keep the lumen patent during sleep. Evidence from a randomised controlled trial suggests that women, patients with a slimmer neck and anterior pharyngeal wall collapse are most responsive (30). Currently, the results of the TESLA-home trial are delayed due to the COVID-19 pandemic, but initial experience in the domiciliary setting suggests that TESLA can be offered as a second line therapy to patients who have previously failed CPAP therapy (31).

#### Surgical Treatments/Maxillofacial and ENT Surgeons

Surgical options are frequently considered in paediatric sleep medicine due to enlarged tonsils. However, in adults they are typically only recommended with enlarged tonsils, adenoids, polyps of significant deviation from normal anatomy for patients unable to tolerate CPAP therapy or those continuing to experience symptoms despite adherence to therapy (32). There are various established and numerous experimental surgical approaches. We list here the more common procedures:

#### Uvulopalatopharyngoplasty(UPPP)/ENT Surgeons

UPP can be offered to patients with mild to moderate OSA, it has shown an overall success rate of about 50%. However, the European Respiratory Society (ERS) recommends patients undergoing the UPPP procedure to be carefully selected with the obstruction caused by OSA being limited to the oropharyngeal area only (21).

#### Tonsillectomy/ENT Surgeons

The ERS task force for non-CPAP therapy recommends this as a single intervention for adults suffering from OSA as a result of tonsillar hypertrophy. Although rare in adults, in children adenotonsillar hypertrophy is a very common cause and adenotonsillectomy can cure these children (21).

#### Maxillomandibular Advancement/Maxillofacial Surgery

Maxillomandibular advancement (MMA) consists of advancement of both the maxilla and the mandible, provided to patients with obstruction in either their hypopharynx or the tongue base, in an attempt to enlarge their retrolingual and retropalatal airway. However, this procedure is invasive and requires approval from a variety of clinicians, not the least the maxillofacial surgeon (21).

Other possible surgical treatments that may be used to treat OSA are listed in the below: (21).

- Nasal surgery
- Radiofrequency surgery (e.g., of tonsils or tongue base)
- Hyoid suspension
- Tongue base and hypopharynx: partial tongue base resection
- Lingual plasty
- Tongue base suspension.

#### Drug Therapy/Pharmacist, Sleep Expert and Research Trials

According to the current ERS guidelines drug therapy is not recommended as a treatment for OSA (21). However, more recently NICE have been appraising the clinical and cost-effectiveness of Pitolisant hydrochloride (a histamine H3-receptor anatagonist) and Solriamfetol (a dopamine and noradrenaline reuptake inhibitor), both stimulant medication, for treating excessive daytime sleepiness caused by obstructive sleep apnoea (33–38). In a more experimental phase is the Atomoxetine plus Oxybutinin (Ato-Oxy) combination. Taken in combination 2 h before sleep has been shown to reduce OSA severity by improving upper airway collapsibility, increasing breathing stability, and augmenting the threshold for arousal in OSA. These effects lead to a reduction in the AHI and an increased genioglossal muscle responsiveness (39).

## Multidisciplinary Team Approach

CPAP is a highly effective therapy to treat OSA and the best available first line treatment. However, patients with OSA require review of their compliance and adherence. In recent analyses of “big data” on a sample of 2.6 mio US citizen the 90-day adherence rate, as defined by >4 h usage of CPAP for more than 70% of the night, was 75% (15). In another sample, the data of about 800,000 patients suggested an age and gender dependant effect on 90-day adherence, ranging from about 51.3–80.6%, with an average adherence of 72.6% (16), and following patients at 1-year and longer sees CPAP adherence rates significantly drop further (40). Whatever the adherence rates in other sleep services, about one in four patients does not receive sufficient treatment at 3-months, and over time the proportion of untreated patients is growing.

**Figure 2:**
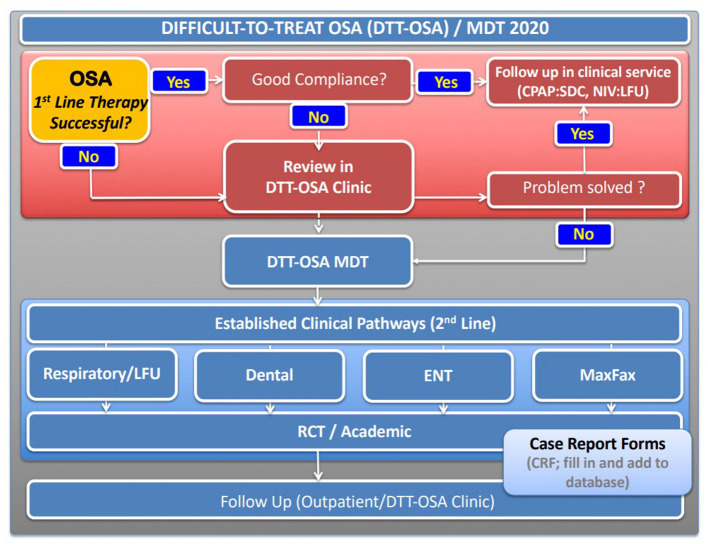
Proposed schematic pathway established at Guy's and St Thomas' NHS Foundation Trust, London for a “difficult-to-treat” (DTT) OSA pathway with a multidisciplinary team meeting (MDT) to discuss 2^nd^ line therapies. Patients who successfully use first line therapy, typically primary airway therapy (CPAP, MAD), remain in the standard clinical follow up. Those who do not manage to control symptoms with standard therapy or achieve sufficient compliance are reviewed in clinic and consented for the discussion at an MDT where individual cases will be presented and followed. RCT, randomised controlled trial.

It is therefore important to consider, establish and develop additional options for non-CPAP therapy to have the choice with the patient, improve adherence and long-term motivation, as significant numbers of patients will otherwise not be sufficiently treated by CPAP. Highly-frequented CPAP services need to consider what therapeutic options they can offer to those who do not use CPAP in the long-term. Bringing together a multidisciplinary team of respiratory physicians and CPAP technologists with dietitians, bariatric services, ENT departments, dentists, maxillofacial surgery and clinical trials units to offer a spectrum of non-CPAP services is essential (Figure 2). In addition, it is important to consider collaborations with the obstetrics department, as pregnant women are also at increased risk of developing OSA. Access to primary airway therapy for pregnant women could help to avoid ill health and adverse outcomes (e.g., pre-eclampsia). Finally, treatment targets for OSA in paediatric cohorts may be more focused on the adenotonsillectomy.

The development of a more structured and systematic approach toward the “difficult-to-treat” patient with OSA, someone who has failed primary airway therapy, is to embed case discussions within a clinical governance framework of a multidisciplinary team (Figure 2). Such an approach should involve all relevant specialties, with clinicians and physiologists involved in the delivery of the sleep service. Under audit conditions, this approach facilitates the prospective provision of evidence-based treatments and the optimal delivery of alternative treatment pathways for patients in whom the first line therapy was unsuccessful. A multidisciplinary approach is standard in most other chronic conditions of non-communicable diseases and there is evidence that team decisions together with the patients provide better outcomes and more individualised care than leaving the decision to individuals.

## Conclusion

Treatment of OSA is moving toward a more bespoke approach, involving the patient and managing long-term ill health. This, however, implies a move away from a “one-size-fits-all” approach. In selected cases, this may mean that non-CPAP therapy may be a better long-term solution to control symptoms and cardiovascular risks than CPAP therapy, despite its proven safety and efficacy. The recent reviews of the evidence by NICE and the new ERS guidelines provide an opportunity to investigate the development of innovative treatment pathways. Systematic observation of clinically relevant outcomes and the follow-up of patients on non-CPAP treatments remains essential. Clinicians need to reassure patients who fail first line therapy in that they are not discharged from clinical services once established treatments has been trialled unsuccessfully. Symptom control is important for patient-centric care and many long-term problems in the management of OSA can be addressed with more tailored solutions, and having the choice of several non-CPAP treatments can make a big difference in the individual case where standard care has not offered successful resolution.

## Author Contributions

All authors have equally contributed to the idea, drafting, and final corrections of this manuscript.

## Conflict of Interest

JS is named inventor on a patent held by Guy's and St Thomas' NHS Foundation Trust and King's College London for an apparatus using electrical stimulation to treat OSA (WO2016124739A1). The remaining author declares that the research was conducted in the absence of any commercial or financial relationships that could be construed as a potential conflict of interest.

## Publisher's Note

All claims expressed in this article are solely those of the authors and do not necessarily represent those of their affiliated organizations, or those of the publisher, the editors and the reviewers. Any product that may be evaluated in this article, or claim that may be made by its manufacturer, is not guaranteed or endorsed by the publisher.
